# Lumbotomy for multicystic dysplastic kidney: A trap for the unwary

**DOI:** 10.4103/0971-9261.72441

**Published:** 2010

**Authors:** Anindya Chattopadhyay, Biswanath Mukhopadhyay, Soumen K. Mitra

**Affiliations:** Department of Pediatric Surgery, NRS Medical College and Hospital and Apollo Gleneagles Hospitals, Kolkata, West Bengal, India

**Keywords:** Horseshoe kidney, lumbotomy, multicystic dysplasia

## Abstract

This is a report of a case of multicystic dysplastic half of a horseshoe kidney (HSK) at surgery for multicystic kidney. During the surgery, through a lumbotomy approach, there was inadvertent injury to a lower polar artery and the pelvis of the normal contralateral half of the HSK, leading to a stormy postoperative course. This report emphasizes the need for accurate preoperative diagnosis before embarking on a lumbotomy, and also reviews the entity of multicystic dysplasia in one half of a HSK.

## INTRODUCTION

Lumbotomy has increasingly become popular for surgery on select pediatric renal conditions. Adequate exposure, ease of making and closing the incision and avoidance of muscle cutting and phantom hernias are the advantages.[1–4] However, the inability of the incision to provide wide exposure makes accurate preoperative diagnosis absolutely mandatory, with malignancies and abnormalities of rotation being contraindications to the approach.[1] We report an unusual case of multicystic dysplastic kidney (MCDK) in one half of a horseshoe kidney (HSK) where inadvertent damage to the contralateral normal pelvis and renal vasculature resulted from the inability to achieve a wider view of the unexpected operative findings.

## CASE REPORT

A 6-month-old male infant with an antenatal diagnosis of right-sided hydronephrosis was referred to us. There was a history of culture proven urinary tract infection (UTI) in the recent past. The child was active, playful and had normal weight and blood pressures. His abdominal examination was unremarkable. A postnatal ultrasonography (USG) done at 3 months of age showed a normal left kidney and a small right kidney made up of cystic spaces without identifiable renal parenchyma. His hemogram was normal and his renal function tests were within normal limits, but urinalysis showed 10–12 pus cells and proteinurea, with a negative culture. A diethylene triamine pentaacetic acid (DTPA) scan revealed a small right kidney with poor perfusion and function (<9%), with a normal left kidney with features of functional obstruction. A micturating cystourethrogram showed no vesico-ureteral reflux (VUR) or other abnormality.

A presumptive diagnosis of right multicystic kidney (MCDK) was made, and in view of the recent UTI, a decision to remove the MCDK was taken via a lumbotomy approach.

At surgery, a cystic small right kidney was encountered with no clearly identifiable renal vasculature. In the course of dissection, it was realized that the lower pole of the kidney was adherent medially and further dissection was required to deliver the kidney. During this dissection, a medium-sized vessel needed division and a normal-looking pelvis was seen and inadvertently injured. Realizing that the anatomy was complicated and the exposure less than adequate, the lumbotomy was closed and a transperitoneal incision was made. On exposing the retroperitoneum, it was seen that the MCDK was part of a HSK with a normal left segment. The lower pole of the left kidney was ischemic – the previously divided vessel was a lower polar artery to this segment. The left pelvis had a rent and this was repaired over a stent brought out as a nephrostomy. The right MCDK was excised and the isthmus was sectioned and the specimen removed. The abdomen was closed with drainage.

Postoperatively, the child went into acute renal shutdown, with minimal output from the nephrostomy and the drain. The child was managed by restriction of fluids. He developed severe hyperkalemia (K^+^:8.8 meq/L) with a rising blood urea and creatinine. An emergent USG with Doppler revealed no flow to the lower pole of the left kidney with a patent left renal artery, fullness of the left pelvis with echogenic debris within it.

Anticipating the need for peritoneal dialysis, and in view of the fullness of the pelvis, the child was re-operated and the nephrostomy was removed, a retrograde double-J (DJ) stent was placed and a Tenchkoff peritoneal dialysis catheter was inserted. Over the subsequent 48 h, the rising creatinine stabilized, hyperkalemia was controlled and the child started producing urine, which drained via the drain and the DJ stent. He developed hypertension and needed Nifedepine for control. Over the next week, his condition stabilized and renal functions returned to normal, permitting removal of the drain and Tenchkoff catheter. He was discharged after 2 weeks, with the indwelling DJ stent, on Nifedepine and urinary antibiotics.

After 6 weeks, the DJ stent was removed and the antihypertensive medications were tapered off. Presently, the child is stable, is growing well, has normal renal functions and is normotensive.

## DISCUSSION

MCDK in one half of a HSK is a rare anomaly, with only 19 cases reported.[5,6] Ascertaining the diagnosis requires the use of cross-sectional imaging techniques such as computed tomogram (CT) or magnetic resonance (MR) scans,[5] studies that are not routinely used in the work-up of patients with a suspected MCDK.

Although USG can diagnose the presence of MCDK in one half of a HSK,[7] the investigation is operator dependant and subtle clues such as malrotation of the contralateral segment may be missed, as in this case. A dimercaptosuccinic acid (DMSA) scan reveals absence of function in the affected segment, but it may or may not indicate the presence of an HSK.[5]

The possibility of MCDK in an HSK may be suggested by the presence of a palpable mass close to the midline, a USG report suggesting abnormal rotation and fusion along the lower renal poles or a DMSA scan suggesting polar fusion.[5] The condition can be confirmed using a cross-sectional imaging study such as CT[8] or MR,[5] which also provides information on the degree of involvement of the isthmus and contralateral kidney.

Because of the proximity of the vessels of the normal segment and the possibility of damage to the great vessels, small MCDKs in HSK may be left alone and managed conservatively.[6] Surgery is indicated in the presence of recurrent infections, pain or the presence of a palpable mass,[9] although, as more information on this rare combination becomes available, expectant management, as in unilateral MCDK, should become the norm.

Surgery for MCDK is often performed through a lumbotomy incision. The incision provides quick access, does not divide any muscles and is easy to close. Pyeloplasty, pyelolithotomy, nephroureterectomy, ureterostomy and heminephrectomy have all been performed through this incision with excellent results, and the safety and efficacy of the approach has been well documented.[1–4]

However, the surgical approach in MCDK in HSK must be transperitoneal to afford exposure to the entire HSK with its vasculature, to allow a safe dissection and excision of the nonfunctioning segment.[5,10] A lumbotomy incision with its relatively limited exposure is hazardous in these cases and can lead to complications, as encountered in this case. Retroperitoneoscopy or laparoscopy may be used. Surgeons may feel guilty of using a large incision to hunt for a small dysplastic kidney. Lumbotomy approach is not good for searching a small kidney. Thus, although rare, the possibility of MCDK in an HSK must be kept in mind before deciding on the approach. A diligent review of the investigations and fresh ones if necessary can spare the surgeon and the patient from a preventable catastrophe.
